# Determination of key structure–activity relationships in siRNA delivery with a mixed micelle system^☆^^☆☆^

**DOI:** 10.1016/j.jconrel.2013.10.013

**Published:** 2013-12-28

**Authors:** Marta Omedes Pujol, Daniel J.L. Coleman, Christopher D. Allen, Olaf Heidenreich, David A. Fulton

**Affiliations:** a Chemical Nanoscience Laboratory, School of Chemistry, Newcastle University, Newcastle upon Tyne, UK; b Northern Institute of Cancer Research, Paul O'Gorman Building, Medical School, Newcastle University, Newcastle upon Tyne, UK

**Keywords:** AML, acute myeloid leukaemia, PNP, polymeric nanoparticle, P1, PCL-*b*-PDMAEMA, P2, PCL-*b*-PEG, siRNA, short interfering ribonucleic acid, NR, Nile Red, FACS, fluorescent activated cell sorting, LSCM, laser scanning confocal microscopy, siRNA, Polymer micelles, Leukaemia, Structure–activity relationships, Gene knockdown

## Abstract

Short interfering ribonucleic acids (siRNAs) offer a highly specific and selective form of therapy for diseases with a genetic component; however the poor pharmacokinetic properties of the molecule have impeded its development into a therapeutic for use *in vivo*. Several different approaches have been taken to develop a successful siRNA delivery system but these systems lack the flexibility for easy optimisation. Here, we propose a polymeric nanoparticle (PNP) system consisting of two amphiphilic diblock copolymers which allow for the rapid determination of structure–activity relationships involving gene knockdown and toxicity. The diblock copolymers self-assemble into monodisperse micelles of defined hydrodynamic diameters ranging from 30 to 100 nm dependent on the copolymer ratio. A luciferase-based high throughput assay varying PNP composition, concentration and siRNA concentration allowed the rapid identification of efficient PNP formulations for adherent and suspension cell lines. Optimised PNPs efficiently knocked down a fusion oncogene in hard to transfect human leukaemic cells raising the possibility of targeting malignant cells in a cancer-specific fashion. This approach allows the optimum PNP formulation to be identified for different cell types and conditions.

## Graphical abstract

**Figure:**
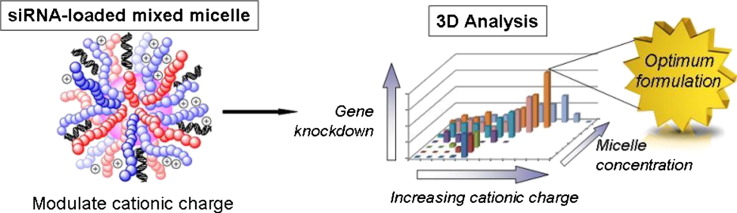

## 1 Introduction

Small interfering RNAs (siRNAs) are short double stranded RNAs which can silence the expression of specific genes by inducing cleavage and subsequent degradation of messenger RNA [1–3]. siRNAs are a very attractive tool to interrogate gene function and hold substantial therapeutic promise, in particular with cancer-specific fusion genes, which are unique to tumour cells and absolutely required for cancer maintenance both in cell culture and *in vivo* [4]. Of particular interest to us is *RUNX1*/*ETO*, a fusion gene found in Acute Myeloid Leukaemia (AML) cells and which acts as a regulator of transcription without intrinsic enzymatic activity, making it a difficult target for conventional drug development programmes, but an ideal target for siRNA therapy [5,6].

The unfavourable *in vivo* pharmacokinetic properties of siRNAs have, however, prevented the realization of their full therapeutic potential [7]. The most limiting factor for the development of siRNA therapies is the safe and efficient intracellular delivery to diseased tissues [8]. Numerous systems have been developed with the aim of overcoming the problem of effective siRNA delivery including liposomes [9,10], cell penetrating peptides [11,12] and polymer nanoparticle-based systems [13]. An often overlooked deficiency of most reported nanoparticle delivery systems is that they cannot be adapted to different cell types in a time- and cost-effective manner; crucial requirements, as it is likely that a delivery platform must be tailored towards its target diseased tissue types. Apart from a few notable exceptions [14–16], detailed structure–activity relationships for siRNA delivery platforms are absent from the literature. This is puzzling when considering their importance in developing and optimising a platform towards a target cell/tissue type. Thus, there is an urgent need for a highly versatile delivery platform, whose structural features can be rapidly and systematically modified, to allow the determination of structure–activity relationships required for tailoring towards a specific tissue.

We chose a cationic polymeric nanoparticle (PNP) approach for siRNA delivery. These nanoparticles possess multiple positive charges for electrostatic interaction with negatively charged siRNA. Therefore, the degree of cationic charge displayed by a siRNA delivery platform is a structural parameter of crucial importance. Adequate levels of cationic charge are required by the PNP to ensure the complexation of siRNA cargos, promote adequate levels of PNP–cell surface interactions and ultimately, cell transfection [17]. Unfortunately, high levels of cationic charge have also been shown to correlate well with toxicity [15], and it is thus vitally important that the level of cationic charge can be optimised to ensure adequate levels of siRNA complexation and transfection with minimal toxicity. In this manuscript we demonstrate by means of a highly insightful three-dimensional analysis, how a simple polymer mixed micelle [18] nanoparticle (PNP) siRNA delivery platform can be exploited to rapidly obtain two key structure–activity relationships in siRNA delivery: how the overall degree of cationic charge displayed by the nanoparticles relates to i) levels of gene knockdown *in vitro*, and ii) levels of cell toxicity.

## 2 Materials and methods

### 2.1 Materials, polymer synthesis, PNP preparation and characterization

Details are given in the Supplementary data.

### 2.2 Gene knockdown using the firefly luciferase reporter assay

Luciferase gene expression was measured using an optimised Luciferase assay. PNP solutions were prepared at: i) different proportions of cationic polymer (0–90% of P1), ii) different overall polymer concentrations and iii) different siRNA (siGL3 or siAGF1) loading concentrations.

#### 2.2.1 293T cells

Passaged 293T cells were seeded in 96-well plates. When the cells reached 70–80% confluency, these were washed with PBS and fresh DMEM D6171 with 10% FBS (150 μl) and a PNP solution (50 μl) was added to each well.

#### 2.2.2 SKNO cells

150 μl of SKNO cells in media at a concentration of 0.67 × 10^6 ^cells ml^− 1^ was loaded in each well of 96 well plates. To each well 50 μl of a PNP solution was added to reach a final concentration of 0.5 × 10^6^ cells ml^− 1^.

The cells were incubated at 37 °C, 5% CO_2_, in a dark and humidified environment for 24 h. 25 μl of 15% luciferin solution (Promega) in media was added to each well. Luminescence was monitored with an Omega FluoStar plate reader at a 4000 gain after 3, 5 and 10 min of the addition of the reagent. The luciferase silencing efficiency was determined by the relative luminescence of treated cells compared to untreated cells (Blank).

### 2.3 Cell association assay (FACS)

Cell association experiments were performed using PNPs loaded with Nile Red (NR), a hydrophobic fluorochrome that can be easily hosted in the hydrophobic core of the micelles. Briefly, block co-polymers P1 and P2, at the appropriate molar ratio (3.3 × 10^− 4^ mmol polymer), were dissolved in *N*,*N*-dimethylformamide (DMF) (0.5 ml), a solvent which both co-polymer blocks are soluble in. Then, 200 μl of Nile Red solution (1 mg/ml in DMF) was added, and the mixture was stirred for 5 min. Deionised water (3.5 ml) was added dropwise at a ratio of 0.04 ml/min with vigorous stirring to promote PNP formation and the NR encapsulation. This solution was transferred into a dialysis tube MWCO 3200 Da and dialysed three times against water for 24–36 h. The volume of water was adjusted to 5 ml and subsequently filtered to achieve a polymer stock solution of 6.7 × 10^− 2^ mM. PNPs prepared in this way showed similar characteristics to particles prepared by sonication (Fig. S7).

293T cells were cultured to 80–90% confluency in 12 well plates. Prior to the transfection, cells were washed with PBS (500 μl) and fresh media (750 μl) was added. PNP solutions with the appropriate siRNA MR loading and polymer concentration were prepared and added to each well (250 μl). After 4 hour incubation, the medium was removed and the cells were washed twice in PBS, trypsinised and harvested with 1 ml of fresh PBS for FACS analysis. In the flow cytometry measurements, the logarithmic fluorescence intensity of untreated cells was set between 10^0^ and 10^1^, and all these cells with intensities higher than 10^1^ were considered to be positive (M1 zone), or cells that have experienced association with the PNPs.

### 2.4 Cell imaging (imaging flow cytometer)

Imaging experiments using an imaging flow cytometer (Amnis ImageStream) were performed to evaluate the cellular internalization of these polymer nanoparticles (PNPs), their siRNA cargos and their localisation within the cell. PNPs were prepared as described above to encapsulate NR (NR; λ_ex_ = 530 nm, λ_em_ = 615 nm); and these were subsequently loaded with a 1:9 MR of Cy5-siAGF1/siAGF1 solution (Cy5; λ_ex_ = 646 nm, λ_em_ = 662 nm).

293T cells (50% confluent) were seeded in a 12 well plate in 1 ml of media. When the cells reached 85–90% confluency, the media was replaced by 750 μl of fresh media, and 250 μl of 8 μM PNP50 at a 1:4 siRNA/polymer molar ratio were added to each well. After 24 h each of these samples was trypsinised and washed twice with PBS. The cells were fixed with a 0.5% PFA solution, washed three times with PBS, and the cell nuclei were counterstained with DAPI (2 μg/ml PBS solution) for 15 min. The samples were further washed twice to remove the excess of DAPI, re-suspended in 200 μl of PBS and analysed in the imaging flow cytometer. The positive samples containing all three fluorochromes (NR, Cy5 and DAPI) were used to set up each channel and the laser intensities. Images from samples of cells containing each individual fluorochrome (colour control) were also collected in order to facilitate compensation between channels, allowing spectral resolution of fluorochromes with overlapping emission spectra. Images were acquired for 1000 cells of each sample. After selecting single and spherical cells and excluding all these out of focus cells, a total of 211 cells were submitted to analysis. The spot counting was achieved by using the IDEAS software, allowing thus statistical analysis to be performed.

### 2.5 Cell imaging (LSCM)

Higher resolution images were obtained by using life imaging on a Nikon A1R (invert) laser scanning confocal microscope (LSCM). 293T cells were seeded into 35 mm diameter glass bottom petri dishes (MatTek) and cultured to reach 50% confluency. Fresh media (1.5 ml) and a solution of PNP50 loaded with Cy5-siAGF1 at a 1:4 siRNA/polymer ratio that was added to a final polymer concentration of 2 μM (500 μl) were added. After 4 h of incubation the media was replaced by fresh one containing Hoechst 33342 nuclear staining (Invitrogen) at a concentration of 2 μg/ml and incubated for further 15 min. The cells were washed twice with PBS and imaged at a × 40 magnification.

### 2.6 Cell viability assay

Cytotoxicity was assessed using the WST-1 assay, a colorimetric enzyme activity assay (Roche Bioscience). PNP solutions in medium were prepared at different proportions of cationic polymer (0–90% of P1), at different overall polymer concentrations (0.4–4 μM), and at different siRNA (siGL3 or siAGF1) loading concentrations (50 nM, 150 nM and 800 nM) analogously to the reporter gene assays in 2.11. Passaged 293T cells were seeded in 96-well plates. When the cells reached 80–90% confluency, these were washed with PBS and fresh media (75 μl) and each specific PNP solution (25 μl) was added to each well. The cells were incubated at 37 °C, 5% CO_2_, in a dark and humidified environment for 24 h. 10 μl of WST-1 reagent was then added to each well and, after 2 h, the absorbance was measured on an Omega FluroStar plate reader at a λ = 455 nm *versus* 655 nm.

## 3 Results and discussion

### 3.1 Polymer nanoparticle design and characterization

**Fig. 1:**
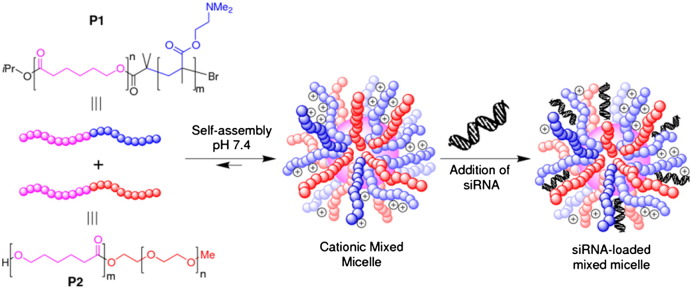
Design of a mixed micelle delivery system, formed from self-assembly of diblock copolymers P1 and P2. These polymers share the same hydrophobic block, polycaprolactone (pink block), which forms the core of the micelle, but differ on their hydrophilic blocks; a cationic PDMAEMA block in P1 (blue block), and a 5 kDa polyethylene glycol (PEG) block in P2 (red block) forming the outer shell of the micelle. The cationic block is decorated with amino functions, partially protonated at physiological pH, which allows the electrostatic interaction with the negatively charged siRNA. PEG, a slightly longer block, provides shielding and stability to the system. This mixed micelle delivery system is highly versatile. By simply varying the molar ratio P1/P2, leads to a rapid optimisation of the overall cationic charge within the particle, which aids its abilities to deliver siRNA tissue/cell specific.

The PNP platform exploits the utilisation of a mixed micelle design strategy [18] (Fig. 1), providing a remarkably rapid and versatile approach for synthesis, with the capacity for structural adaptation. The PNPs are prepared from the self-assembly of two different amphiphilic diblock copolymers (P1, P2) into a stable mixed micelle which can be considered a polymeric nanoparticle. Both P1 and P2 possess hydrophobic blocks of similar M*_w_* of 4.5 kDa based upon biocompatible poly(caprolactone) (PCL), but differ in their hydrophilic blocks, with P1 possessing an 15.5 kDa poly((dimethylamino)ethylene methacrylate) (PDMAEMA) block which is partially protonated under physiological conditions [14,19,21], and P2 possessing a 5 kDa PEG block resulting in total molecular weights of 9.5 kDa and 20 kDa for P1 and P2. The partially protonated amine groups in P1 will complex siRNA cargo and possess sufficient buffering capacity to promote endosomal rupture through the ‘proton sponge effect’ [22], facilitating endosomal escape into the cell. The function of PEG block, if suitably long enough, is to provide colloidal stability to the micelle, and to shield the siRNA cargo, thus increasing its *in vivo* circulation times. Importantly, the net cationic charge displayed by the PNP can be modulated simply by changing the molar ratios of P1:P2. This mixed micelle approach negates the need for extensive and time-consuming resynthesis of PNPs as, unlike triblock copolymers (ABC or ABA) [23–25], and single diblock copolymers (AB) [26,27], the two independent amphiphilic diblock copolymers (AB and CB) open the possibility of constructing rapidly numerous mixed micelle compositions.

**Fig. 2:**
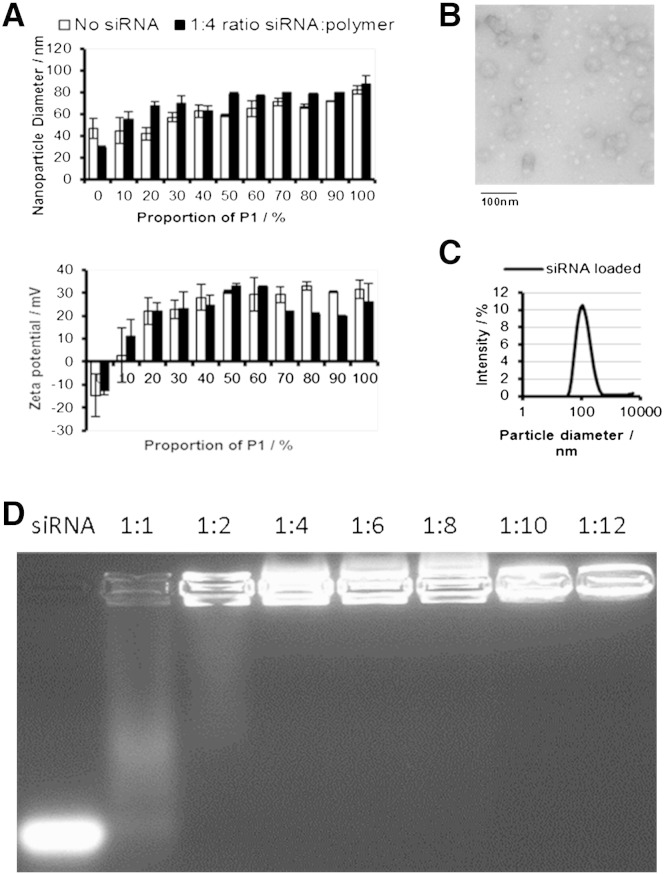
A) Bar charts showing how particle diameter (top) and ζ-potential (bottom) change as the proportion of P1 within the PNP increases. The PDIs of PNPs were all < 0.3 B) TEM images of PNP50 loaded with a 1:4 MR siRNA/polymer. C) A typical particle size histogram for a PNP (PNP50) loaded with siRNA displayed by dynamic light scattering analysis (PDI = 0.2). D) Gel electrophoresis of PNP50 loaded with different ratios of siRNA/polymer. siRNA appears to be fully incorporated into the complex at a ratio 1:4 with total polymer. Naked siRNA was used as a control.

Eleven PNPs were prepared (Table 1), in which the molar ratio of P1:P2 was changed from 100:0 to 0:100 in 10% intervals to afford PNP0–PNP100, where the number indicates the percentage of cationic polymer P1 chain within the mixed micelle. Dynamic light scattering (DLS) indicated the formation of nanoparticles with hydrodynamic diameters ranging from 30 to 80 nm, which size is in the appropriate range to allow the exploration of enhanced permeability and retention effects (Fig. 2A, top). Loading of this series of PNPs with siRNA in a 1:4 molar ratio of siRNA to polymer (P1 + P2) increased the hydrodynamic diameter by ~ 20%, implying a degree of PNP swelling upon siRNA complexation. TEM analysis (Figs. 2B, S8) of PNP50 loaded with siRNA confirmed discrete spherical nanoparticles with diameters of 40–50 nm, which is smaller than the hydrodynamic diameters measured by DLS (Figs. 2C, S9). The difference between sizes in DLS and TEM can be attributed to solvation of the coronal hydrophilic polymer chains, resulting in a larger PNP diameter in solution. Both DLS and TEM studies indicate the formation of well-defined spherical nanoparticles, and the hydrodynamic diameter of siRNA-loaded PNPs remained stable over a time period of 40 days, suggesting a substantial shelf-life of these PNPs (Fig. S10).

The degree of net cationic charge present within each PNP was estimated by ζ-potential measurements (Fig. 2A, bottom), which show that the ζ-potential increases as the proportion of P1 increases up to PNP30, after which it plateaus at 30 mV. Interestingly, the same trend is observed when the PNPs are loaded with siRNA. Gel retardation experiments show that a 1:4 ratio of siRNA:polymer shows a good level of siRNA complexation with most formulations containing ≥ 30% P1 (Figs. 2D and S11). When observed in both deionized water and saline PNP50 loaded with a 1:6 ratio of [siRNA]:[P1 + P2] the size and PDI of the PNP did not change significantly, although the ζ-potential of the PNPs in saline was decreased (Fig. S12).

The size of particles was observed in media containing up to 20% serum (Figs. S13–S14, Table S1). Particles appeared to swell by between 20 and 40% of their size in FBS free media and the PDI increased drastically, although this is in part due to the detection of particles present in the serum by DLS.

**Fig. 3:**
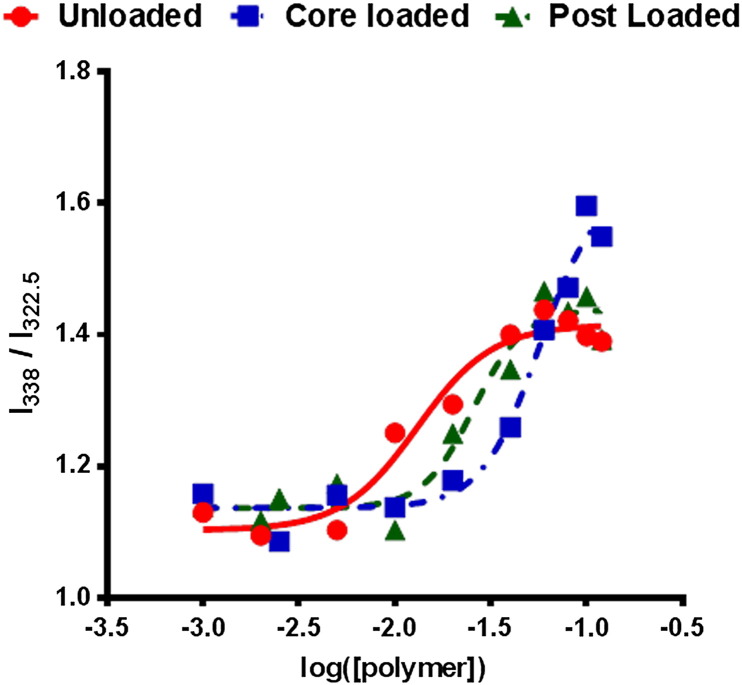
The critical micelle concentration (CMC) of a nanoparticle is the concentration above which any added amphiphilic molecules have a high probability of appearing as micellar aggregates. This factor sets a lower limit on the concentration at which the micelle will be effective. Using the pyrene fluorescence spectroscopy method which utilises the changes in the intensity of the vibrational bands of pyrene depending on the solvent environment by measuring the changes in fluorescence at I_322.5_ (pyrene in a hydrophilic environment) and I_338_ (pyrene in a hydrophobic environment) and plotting the I_338_/I_322.5_ the CMC of PNP50s was estimated by differentiating the point of inflection of the slope and using this equation to calculate where the slope crosses the base line of the curve [28]. The loading of siRNA to the PNP increases the CMC of the particle from 300 nM (circles) to 880 nM (triangles) when siRNA was added after micelle formulation and to 1.7 μM (squares) when added before.

The critical micelle concentration (CMC) indicates the lowest concentration at which micelles and PNPs can form. The CMC for unloaded PNP50 was 300 nM. PNP50 loaded with siRNA after and during micelle assembly gave CMCs of 880 nM and 1.7 μM, respectively, which implies a destabilisation of the micellar structure by the complexed siRNA (Fig. 3). Importantly, these CMC values mark the lower limit of PNP concentration which can be applied *in vitro* and *in vivo* whilst ensuring the integrity of the micelle.

### 3.2 Cell association and internalization

**Fig. 4:**
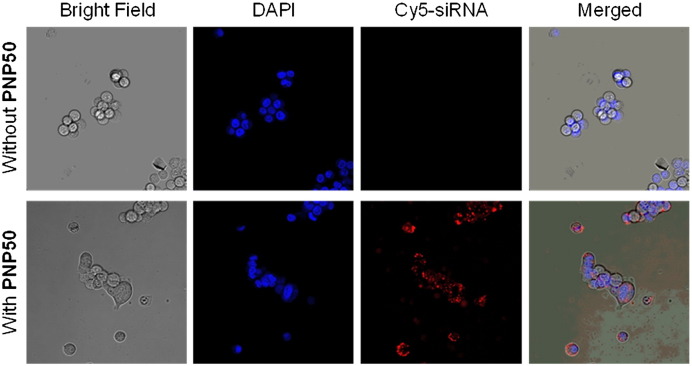
Confocal microscopy images of 293T cells incubated with 700 nM of Cy5-siAGF1/siAGF1 (1:9 MR) loaded onto 2.8 μM PNP50 (bottom row) and without a nanocarrier (top row), using a 40 × magnification.

**Fig. 5:**
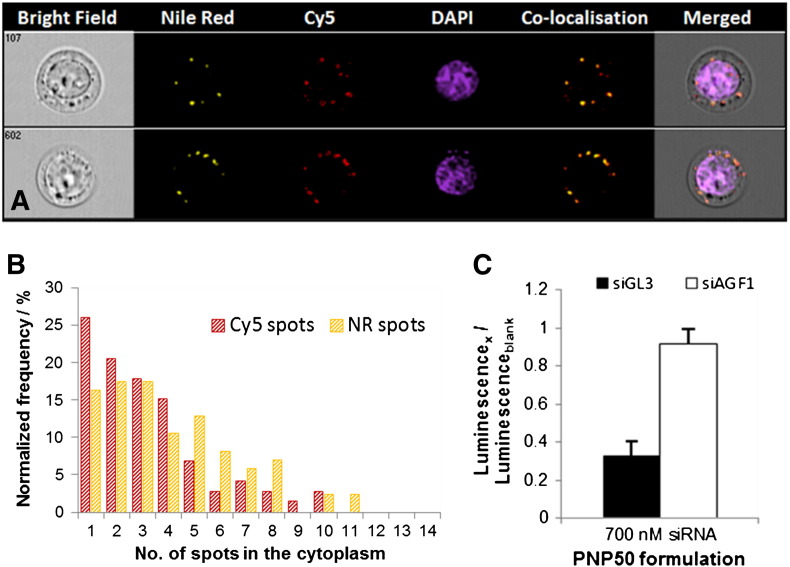
A) Imaging flow cytometry (ImageStream) allowed a cell count and cell imaging to be performed at the same time. Three fluorescent probes, NR (encapsulated in the PNP's hydrophobic core), Cy5 (complexed to the siRNA) and DAPI were observed in three different channels. B) Spot count of vesicles in the cell cytoplasm containing NR and Cy5 showing dye co-localisation. C) Luciferase gene knockdown in 293T cells is presented for both, control siRNA (siAGF1) and luciferase specific siRNA (siGL3).

To better understand the cell association and uptake properties of these PNPs, further experiments were performed in human 293T cells which were analysed using flow cytometry, imaging flow cytometry and confocal microscopy. To that end, we loaded the PNPs to saturation with Nile Red (NR), a highly hydrophobic dye, which is encapsulated within the PCL core of the particle and, Cy5-labelled siAGF1 for tracking of the siRNA cargo. The incorporation of these fluorophores into the PNP did not significantly alter the particle size or zeta potential. Importantly, all experiments were performed in the presence of 10–20% foetal calf serum to mimic *in vivo* conditions. Flow cytometry showed that the PNP concentration, but not the siRNA/polymer molar ratio (*i.e.* the N/P ratio) or the proportion of P1 in the PNPs correlated with cell association (Figs. S15–17). Confocal microscopy and imaging flow cytometry independently showed the presence of NR-positive punctate pattern in the cell cytoplasm within 5 h after addition of siRNA-PNP50 nanoparticles to cells, indicating cellular uptake of the nanoparticles *via* an endosomal route (Figs. 4 and 5A). Importantly, the Cy5 signal is located in the same vesicles as the NR proving co-localisation of PNP and siRNA (Fig. 5B).

### 3.3 Intracellular release and gene knockdown

In order to demonstrate functional cytoplasmic siRNA release we measured the knockdown capacity of siRNA-loaded PNPs. Knockdown experiments were performed with a reporter cell line, 293T SLIEW, which stably expresses the gene coding for firefly luciferase. Cells were treated with PNP50 loaded either with anti-luciferase siRNA siGL3 or a control siRNA siAGF1 targeting the *RUNX1*/*ETO* fusion gene at a molar siRNA to polymer ratio of 1:4. Under these conditions, we observed a very robust (70%) and highly reproducible (p < 0.001) siGL3-specific knockdown of luciferase activity indicating an efficient cytosolic release of functional siRNA. Furthermore, the negative siAGF1 control showed that this suppression of luciferase is due to RNA interference and not to toxicity-related impairment of luminescence (Fig. 5C).

### 3.4 Three dimensional analysis: determination of structure–activity relationship

**Fig. 6:**
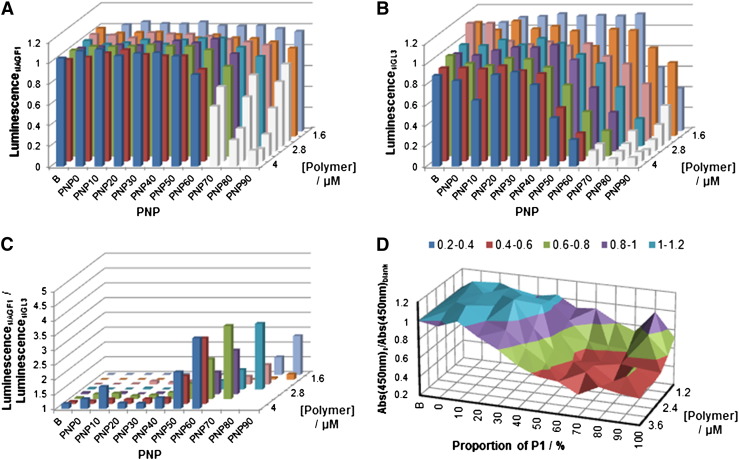
Bar graphs showing data from titrations of the total polymer concentration, proportion of cationic polymer P1 within the PNP and siRNA load of the micelle. A) Gene knockdown in 293T cells treated with PNPs loaded with the control siAGF1. Reductions in luminescence occur as a consequence of toxicity effects. B) Gene knockdown in 293T cells treated with PNPs loaded with the active siGL3, where reductions in luminescence occur as a consequence of both gene knockdown and toxicity effects. C) Normalisation of the these two data sets shows actual gene knockdown, omitting toxicity effects. The formulations with the highest degree of knockdown are represented as the tallest bars. In A) and B) formulations showing toxicity (knockdown of > 20% using siAGF1 loaded PNPs) are shown in grey. D) PNP toxicity determined by WST-1 assay.

Confident that the PNPs are capable of facilitating siRNA uptake and gene knockdown, the SARs between cationic charge and gene knockdown and toxicity were investigated. We applied an assay in which the level of gene knockdown was determined as a function of three variables: i) the cationic charge displayed by the PNP (which is modulated by the ratio of P1:P2), ii) the overall PNP concentration, and iii) the siRNA concentrations. This assay allows for a “three-dimensional” analysis to determine the influence of these three variables on knockdown. To that end we used a luciferase reporter gene in a 96 well plate format to gauge the effect of treatment with PNP:siRNA formulations loaded with the luciferase siRNA siGL3 on cell luminescence. siAGF1, an siRNA targeted to RUNX1/ETO as a control. Any knockdown seen in the cells treated with the control siRNA is on account of toxicity rather than siRNA action (Fig. 6A), whereas knockdown observed in the cells treated with siGL3 must arise on account of both actual knockdown and toxicity effects (Fig. 6B). The effects of siRNA concentration were addressed by performing the assay at three levels of siRNA loading.

The typical 3D plot obtained with 293T SLIEW cells, Fig. 6A, shows that as the proportion of cationic polymer within the PNP increases (x-axis), and as the overall PNP concentration increases (y-axis), the level of gene knockdown on account of toxicity also increases (white bars, Fig. 6A). The analysis was then repeated using siGL3 as cargo. This plot (Fig. 6B) shows a similar trend, with luciferase activity declining as both the proportion of cationic polymer within the PNP and the overall PNP concentration increases. However, unlike the control, siAGF1, the inhibition of luciferase was observed for a larger range of PNP ratios and polymer concentrations. Normalising luminescence of siAGF1 treated cells to those treated with siGL3 for each formulation of micelle affords a 3-dimensional bar graph (Fig. 6C), where the height of each bar represents the level of knockdown whilst accounting for toxicity. For the sake of clarity, those experiments which resulted in unacceptably high levels of toxicities (< 80% luminescence normalised to blank cells) have been omitted. The emergence of a ‘front’ of several formulations which afford similar high levels of knockdown in 293T cells (Fig. 6C) shows that toxicity trails knockdown. This observation implies that the knockdown accomplished by PNPs loading 800 nM siRNA arises on account of siRNA activity. Lowering the siRNA concentration to 200 and 50 nM (Fig. S18) caused an inferior knockdown, and particularly at the lowest concentration, a recession of the “knockdown front” towards lower P2 content caused by increased toxicity when compared to 800 nM. Therefore, a dominant feature of these analyses is that successful formulations for 293T SLIEW cells possess 30–50% cationic polymers, and imply that higher siRNA loadings substantially diminish non-specific inhibition of luminescence and yield higher levels of gene knockdown. These observations also suggest that the free cationic charges of P1 are responsible for the toxicity of the PNP. Independent cell viability experiments validated these findings (Fig. 6D).

### 3.5 Three dimensional analysis of leukaemic cells growing in suspension

**Fig. 7:**
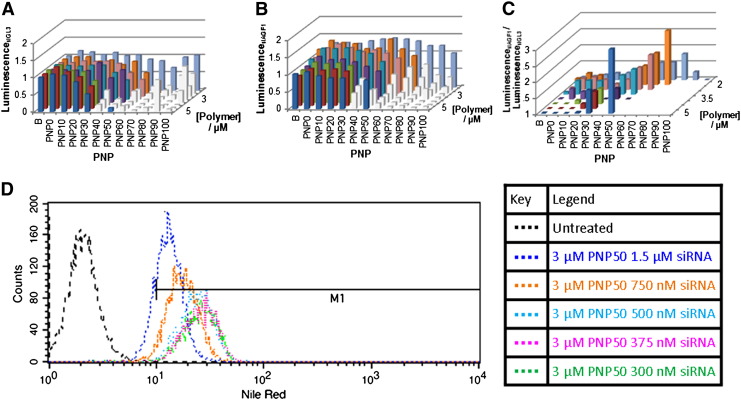
Bar graphs showing data from titrations of the total polymer concentration, proportion of cationic polymer P1 within the PNP and siRNA load of the micelle A) Gene knockdown in SKNO-1 cells treated with PNP loaded with the control siAGF1. Reduction in luminescence occurs as a consequence of toxicity effects. B) Gene knockdown in SKNO-1 cells treated with PNP loaded with the active siGL3, where reductions in luminescence occur as a consequence of both gene knockdown and toxicity effects. The formulations with the highest degree of knockdown are represented as the tallest bars. In A) and B) formulations showing toxicity (knockdown of > 20% using siAGF1 loaded PNPs) are shown in grey. The effectiveness of the lower concentration of siRNA in these cells can be explained by cell association D); as siRNA load increases the association of PNPs with SKNO-1 cells appears to decrease.

These experiments were repeated in SKNO-1 SLIEW cells (Fig. 7), a human AML cell line which expresses the *RUNX1*/*ETO* fusion gene and which as a suspension cell line is notoriously difficult to transfect [20]. This cell line shows a similar trend to the 293T cells in terms of the toxicity of the PNPs decreasing as higher concentrations of siRNA are used, causing the ‘front’ of most active formulations to move towards higher P2 content. In this leukaemic cell line, the knockdown of luciferase is highest at 500 nM siRNA and declined at 1000 nM (Figs. 7C and S19), contrary to the 293T cells where the level of knockdown increases with higher siRNA loading. This difference is likely due to the uptake of the siRNA loaded PNPs into SKNO-1 cells being more dependent on the overall positive surface charge of the PNP, and therefore the neutralisation of this charge with siRNA appears to limit the uptake and resultant gene knockdown. Indeed, unlike the 293T cells, increasing the siRNA:polymer ratio decreases association with SKNO-1 cells (Fig. 7D).

### 3.6 Knockdown of a leukaemic fusion gene with an optimised PNP formulation

**Fig. 8:**
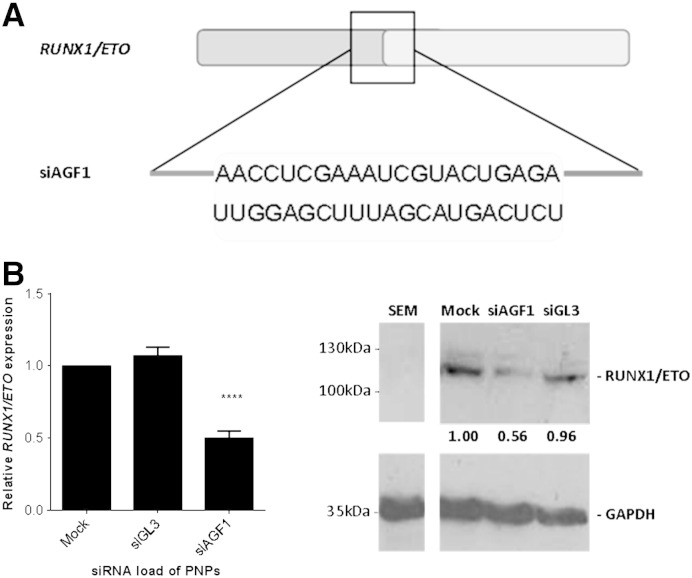
Knockdown of the *RUNX1*/*ETO* fusion gene. A) siAGF1 is complimentary to the fusion gene translocation and, consequently, has no effect on *RUNX1* or *ETO* in healthy cells. B) Knockdown of *RUNX1*/*ETO* mRNA relative to the housekeeping gene GAPDH in SKNO-1 cells treated with 3 μΜ PNP50/500 nM siAGF1 compared to control siGL3 and untreated cells (Mock). ****, p < 0.0001 for n = 9. Error bars indicate SEM. C) Knockdown of RUNX1/ETO fusion protein in SKNO-1 cells treated with 3 μΜ PNP50/500 nM siRNA with siGL3 serving as a non-targeting control. Values represent the relative expression for each sample normalised to GAPDH and relative to untreated cells (mock).

The utility of these 3D analyses was evaluated by post-transcriptional knockdown of the *RUNX1*/*ETO* fusion gene with siAGF1. *RUNX1*/*ETO* is generated by a translocation between the long arms of chromosomes 8 and 21 t(8;21) and is found in 10–15% of cases of AML [29]. siAGF1 comprises an siRNA that is specific to t(8;21) (Fig. 8A) and consequently has no effect on cells lacking the translocation. A 3 μM PNP50 loaded with 500 nM siRNA, which exhibited good gene knockdown and was not shown to be toxic by the 3D analysis, caused a comparable of knockdown of *RUNX1*/*ETO* in t(8;21) positive SKNO-1 cells, both at mRNA level (Fig. 8B), and at protein level (Fig. 8C).

## 4 Conclusion

These 3D plots allow a high throughput screen of PNPs which can be used to identify the optimum drug formulation for a specific set of conditions. This approach will be useful for the further development and selection of formulations to take forward to *in vivo* studies. However, it is also important to note the clear toxicity of the cationic diblock used in this system, which should be considered in the future development of formulations.

This work highlights how a versatile mixed micelle polymer nanoparticle, in combination with the introduction of a 3-dimensional assay, which allows the rapid construction of SARs involving cationic charge and gene knockdown/toxicity. It permits the rapid determination of the optimal level of cationic charge to facilitate knockdown, and the optimal levels of PNP and siRNA loading to achieve prominent gene knockdown in such different systems such as 293 T and SKNO-1 cells and diminishes the expression of the “undruggable” cancer-specific fusion gene *RUNX1*/*ETO*. These particles show promise for the future of siRNA delivery.

## Figures and Tables

**Table 1:** Listing of the eleven PNP constructs and their compositions.

Entry	PNP	Proportion of P1 (%)	Proportion of P2 (%)
1	PNP0	0	100
2	PNP10	10	90
3	PNP20	20	80
4	PNP30	30	70
5	PNP40	40	60
6	PNP50	50	50
7	PNP60	60	40
8	PNP70	70	30
9	PNP80	80	20
10	PNP90	90	10
11	PNP100	100	0
