# Supplementary material for: Determination of key structure–activity relationships in siRNA delivery with a mixed micelle system
Source: J Control Release. 2013 Dec 28;172(3):939–45. doi: 10.1016/j.jconrel.2013.10.013 (PMC3898608; doi:10.1016/j.jconrel.2013.10.013)
Supplement: Supplementary file 1 — Supplementary material. [file mmc1.doc]

Determination of Key Structure-Activity Relationships in siRNA Delivery with a Mixed Micelle Delivery System

Marta Omedes-Pujol^a^, Daniel J.L. Coleman^b^, Christopher D. Allen^b^, Olaf Heidenreich^b^* and David A. Fulton^a^*

**Table of contents**

**Supplementary Materials and Methods**

**Figures**

**– Polymer characterisation**

**Figure S1.** Synthesis of **P1**

**Figure S2.** ^1^H NMR of polymer **P1**

**Figure S3.** GPC trace of polymer **P1**

**Figure S4.** Synthesis of **P2**

**Figure S5.** ^1^H NMR of polymer **P2**

**Figure S6.** GPC trace of polymer **P2**

**– Mixed micelle physico-chemical properties**

**Figure S7.** The effect of Nile Red on particle characteristics

**Figure S8.** Mixed micelle particle size by transmission electron microscopy

**Figure S9.** Mixed micelle particle size by dynamic light scattering

**Figure S10.** Stability of PNP50

**Figure S11.** Gel electrophoresis for siRNA loading

**Figure S12.** PNP50 characteristics in water and saline

**Figure S13.** PNP50 particle size in media and 10% FBS

**Figure S14.** PNP50 particle size in media and 20% FBS

**– PNP biological activity**

**Figure S15.** Cell association at different micelle concentrations

**Figure S16.** Cell association at different siRNA loadings

**Figure S17.** Cell association at different percentage of **P1**

**Figure S18.** 3D gene knockdown assay by luciferase down regulation in 293T cells

**Figure S19.** 3D gene knockdown assay by luciferase down regulation in SKNO-1 cells

**Tables**

**Table S1.** Particle sizes in media

**Table S2.** Confocal microscopy laser settings

**Supplementary Materials and Methods**

- 1. *Materials*

All chemicals were purchased from Sigma-Aldrich or Alfa Aesar and were used as received without further purification. ε-Caprolactone was purified by vacuum distillation at 70 °C after drying overnight over CaH_2_. 2-Propanol was distilled at atmospheric pressure at 85 °C and stored over molecular sieves. All organic solvents (PhCH_3_, THF, CH_2_Cl_2_) were dried and distilled prior to use. Water was purified using a Milli-Q Millipore instrument. The sequences of siGL3 luciferase siRNA 5’-CUU ACG CUG AGU ACU UCG A dTdT-3’ (sense strand) and 5’-UCG AAG UAC UCA GCG UAA G dTdT-3’ (antisense strand) and the sequences of siAGF1 control siRNA were 5’-CCU CGA AAU CGU ACU GAG AAG dTdT-3’ (sense strand) and 5’-UCU CAG UAC GAU UUC GAG GUU dTdT-3’ (antisense strand) were obtained from MWG Eurofins (Ebersbach, Germany), Axolabs (Kulmbach, Germany) or Purimex (Grebenstein, Germany). The concentration of siRNA was determined by measuring the absorbance at 260 nM using a NanoDrop spectrophotometer. ^1^H NMR spectra were obtained in CDCl_3_ using a Bruker Advance 300 or a Jeol 400 spectrometer and analysed using MestrecNova. Chemical shifts are recorded in ppm relative to CHCl_3_.

- 1. *Synthesis of Poly(ε-caprolactone)-b-poly(2-(dimethylamino)ethyl methacrylate)*
     1. *Synthesis of Poly(ε-caprolactone)*

In a flame-dried Schlenk flask under nitrogen, PhCH_3_ (5 ml), tin(II) 2-ethylhexanoate (60 mg, 0.15 mmol) and 2-propanol (17.5 µl, 0.30 mmol) were charged. The mixture was stirred for 10 minutes and ε-caprolactone (1.5 g, 13.14 mmol) was added. The flask was submerged in an oil-bath at 110 °C for 24 hours. The reaction mixture was left to cool to room temperature and the polymer was precipitated twice in 0°C Et_2_O (300 ml). The polymer precipitate was then isolated by vacuum filtration. The filtrate was further dried under high vacuum yielding a white powder (1.46 g), which was confirmed to be polycaprolactone (PCL) (**Fig. S1**, product **1**). ^1^H NMR (300 MHz, CDCl_3_): 5.00 (septet, *J* = 4.8 Hz, 1H), 4.06 (t, *J* = 5.1 Hz, 2H), 2.31 (t, *J* = 5.7 Hz, 2H), 1.70-1.60 (m, 4H), 1.43-1.33 (m, 2H), 1.22 (d, *J* = 4.8 Hz, 6H). The degree of polymerisation can be determined by comparing integration of the (*CH*_3_)_2_CH signal of the end group with the integration of the *CH_2_*OH signal of the PCL block, and was found to be ~40.

- - 1. *Synthesis of Br-terminated poly(ε-caprolactone)*

PCL (1 g, 0.21 mmol) was charged into a 2 neck-round bottomed flask, and the flask was purged with nitrogen. CH_2_Cl_2_ (15 ml) was added and the resulting solution was cooled to 0 °C in an ice/water bath and Et_3_N added. A solution of bromoisobutyryl bromide (3.16 mmol) in CH_2_Cl_2_ (15 ml) was added dropwise over 30 min. The reaction mixture was removed from the ice/water bath, allowed to warm to room temperature and stirred overnight. To the reaction mixture, a solution of saturated NaHCO_3_ (30 ml) was added and stirred for 15 min. The organic layer was separated and the aqueous layer washed with CH_2_Cl_2_ (2 × 10 ml). The combined organic extracts were dried over MgSO_4_ and the solvent was removed under vacuum. The remaining oil was re-dissolved in CH_2_Cl_2_ (4 ml) and this solution was precipitated into 0°C Et_2_O (250 ml), and the solid product collected by filtration. This procedure was repeated twice and the product was dried under high vacuum to yield a white powder (0.654 g) (**Fig. 1**, product **2**). ^1^H NMR (300 MHz, CDCl_3_): 5.00 (septet, *J* = 4.8 Hz, 1H), 4.06 (t, *J* = 5.1 Hz, 2H), 2.30 (t, *J* = 5.7 Hz, 2H), 1.93 (s, 6H), 1.79-1.60 (m, 4H), 1.44-1.34 (m, 2H), 1.23 (d, *J* = 4.8 Hz, 6H).

- - 1. *Synthesis of poly(ε-caprolactone)-b-poly(2-(dimethylamino)ethyl methacrylate)*

A Schlenk flask was charged with 2-(dimethylamino)ethyl methacrylate (1.72 ml, 10.2 mmol) and THF (2 ml). This solution was degassed five times, and the vessel was backfilled with N_2_ and allowed to warm up to room temperature. This solution was transferred to another Schlenk flask containing the macroinitiator (2) (500 mg, 0.10 mmol), CuBr (14.6 mg, 0.10 mmol) and 2,2’-bipyridyl (33.5 mg, 0.21 mmol) under N_2_. The reaction mixture was submerged into an oil-bath at 60 °C and left to stir overnight, during which time it changed colour from brown to green. The reaction mixture was then passed through a basic alumina column washed with THF to remove the copper salt. Solvent was then removed under vacuum and the remaining oil was dissolved in THF (1 ml) and H_2_O (4 ml). The sample was dialysed (dialysis tube MWCO 3,200 Da) for 24 h and freeze-dried to obtain 1.2 g of a white solid **P1** (**Fig. S1**). The ^1^H NMR spectrum and the GPC chromatogram are presented in **Figs. S2** and **S3**.

- 1. *Synthesis of poly(ethylene glycol)-b-poly(ε-caprolactone)*

Monomethylether polyethylene glycol (PEG; 5 kDa) (1 g, 0.20 mmol) was dissolved in PhCH_3_ (5 ml) in a flame-dried Schlenk flask charged with a magnetic stirrer bar under an atmosphere of N_2_. Sn(Oct)_2_ (40 mg, 0.10 mmol) was added and the solution was stirred for 10 min. ε-Caprolactone (0.88 ml, 8 mmol) was added and the reaction was left to stir for 24 h at 110 °C under an atmosphere of N_2_. The reaction mixture was precipitated into ice-cold Et_2_O (250 ml) and the product collected by filtration. The filtrate was dried under high vacuum and to afford 1.72 g of **P2** as a white powder (**Fig. S4**). The ^1^H NMR spectrum is presented in **Fig. S5**. The degree of polymerisation can be determined by comparing integration of the O*CH_2_CH_2_*O signal of the PEG block with the integration of the *CH_2_*OH signal of the PCL block, and was found to be ~37. The GPC chromatogram showing the polydispersity is presented in **Fig. S6**.

- 1. *Gel permeation Chromatography (GPC)*

Gel permeation chromatography (GPC) was conducted on a Varian ProStar instrument (Varian Inc.) equipped with a Varian 325 UV-vis dual wavelength detector (254 nm), a Dawn Heleos II multi-angle laser light scattering detector (Wyatt Technology Corp.), a Viscotek 3580 differential RI detector, and a pair of PL gel 5 µm Mixed D 300 × 7.5 mm columns with a guard column (Polymer Laboratories Inc.) in series. Near monodisperse polystyrene standards (Agilent Technologies) were used for calibration. Data collection was performed with Galaxie software (Variant Inc.) and chromatograms analysed with the Cirrus software (Varian Inc.) and Astra software (Wyatt Technology Corp.)

- 1. *Preparation of PNPs and siRNA loading*

PNPs were prepared by dissolving different molar rations of polymers **P1** and **P2** directly into buffered aqueous solution or deionized water, to achieve a known total concentration of the two polymers ([P1 + P2]). Ultrasonic agitation was used to assist the dissolution of the polymers. The solutions were filtered and diluted with buffered aqueous solution to a final concentration. siRNA was added to afford different [siRNA]:[P1+P2] molar ratios, and the solutions were sonicated for further 30 min.

- 1. *Agarose gel electrophoresis*

A 3% agarose gel electrophoresis assay was performed to evaluate the loading capacity of mixed micelles polymer nanoparticles (PNP). 5 µl of 10,000 × GelRed staining (Biotium) per 50 ml of agarose gel was added to the gel before casting. Samples were prepared to a determined [siRNA]:[**P1** + **P2**] ratio using a 2µM siRNA concentration. 4 µl of 6× DNA loading buffer (Fermentas) was mixed with 20 µl of sample, and samples were loaded into wells. The gel was allowed to run for 1 h at 70 V and visualised with a BioRad transluminator (UV).

- 1. *Dynamic light scattering (DLS) and ζ-potential analysis*

Hydrodynamic diameter (*D*_h_) and ζ-potential of each mixed micelles polymer nanoparticle in various aqueous solutions were determined by dynamic light scattering (DLS) using a MALVERN Zetasizer Nano Series ZS operating at 20 °C with a 633 nm (red) laser module. Measurements were made in triplicate, at a detection angle of 173 ° (back scattering). Samples at a polymer concentration of 1 mg/ml were previously filtered through a 200 nm syringe filter, and placed into disposable cuvettes of 70 µl of capacity.

- 1. *Transmission electron microscopy (TEM)*

Transmission electron microscopy (TEM) studies were performed at ambient temperature using a Philips CM100 transmission electron microscope with Compustage and high resolution digital image capture. Samples were negatively stained using the single droplet procedure. Briefly, a drop of 20 µl of a 2.8µM total polymer PNP loaded with 700 nM siRNA was placed onto a 400 mesh copper EM grid. Each solution was washed twice with water followed by negative staining (2% (w/v) uranyl acetate in water). Excess sample stain was removed carefully by touching the grid edge to the edge of a filter paper wedge.

- 1. *Critical micelle concentration (CMC)*

The critical micelle concentration (CMC) of PNPs was determined by pyrene fluorescence method as previously described. A solution of pyrene in acetone (15 µM) was aliquoted into a series of glass vials wrapped in aluminium foil and the acetone was evaporated. A PNP50 stock solution in deionised water (2 mg/ml, 0.13 mM) was prepared. A series of 5 ml PNP solutions (or 1 ml when siRNA (1:4 MR siRNA/polymer) was loaded) in deionised water were prepared to a total polymer concentration ranging from 1 – 0.002 mg/ml to afford a final pyrene concentration of 670 nM. Samples were incubated for 24 h at 37 °C in the dark with stirring. The excitation spectra (300 - 360 nm) of each sample were recorded at 20 °C on a Digilab F-2500 Fluorescence Spectrophotometer (Hitachi) with an emission wavelength of 390 nm and slit widths of 2 nm. The fluorescence intensity ratio of I_338_/I_332.5_ was analysed as function of the log_10_ of total polymer concentration.

- 1. *Cell culture*

The human embryonic kidney cell lines 293T and 293T SLIEW, the latter lentivirally transduced for stable luciferase expression, were cultured in DMEM (Dulbecco’s modified Eagle’s medium) D6171 supplemented with 2 mM L-Glutamine and 10% Fetal Bovine Serum (FBS). Cells were incubated at 37 °C, 5% CO_2_, in a dark humidified environment and passaged when they reached confluency (every 2 to 3 d).

The human leukaemic cell line SKNO-1 SLIEW (SKNO-1 cells transduced for stable luciferase expression), were culture in RPMI 1640 (Gibco) supplemented with 7 ng/ml GM-CSF (granulocyte, macrophage colony stimulating factor) and 20% FBS. Cells were incubated at 37 °C, 5% CO_2_, in a humidified environment and split down to 0.5
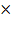
10^6^ cells ml^-1^ every 3 d.


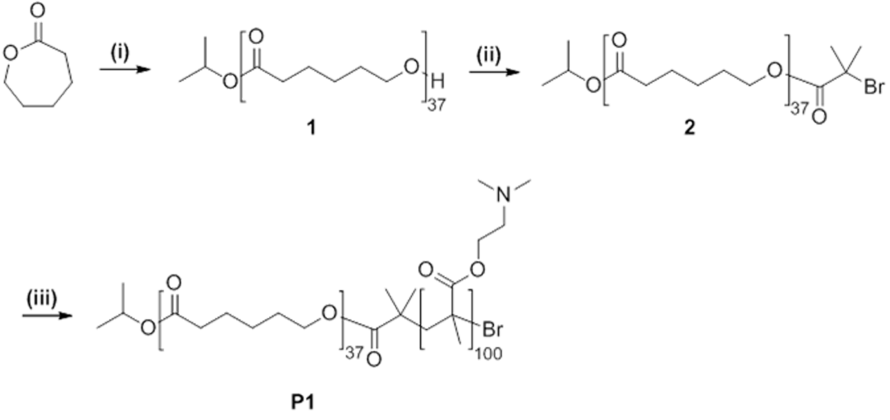


**Fig. S1** – Synthesis of poly(ε-caprolactone)-*b*-poly(*N*,*N*-(dimethylamino)ethylmethacrylate) **P1**: (i) 2-propanol, Sn(Oct)_2_, PhCH_3_, 110 °C, 24 h. (ii) Bromoisobutyryl bromide, NEt_3_, CH_2_Cl_2_, 0 °C to rt, 18 h. (iii) 2-(Dimethylamino)ethyl methalacrylate, CuBr, 2,2’-bipyridyl, THF, 60 °C, 18 h.

**Figure S2.** ^1^H NMR spectrum (400 MHz, CDCl_3_) of diblock copolymer **P1**.

**
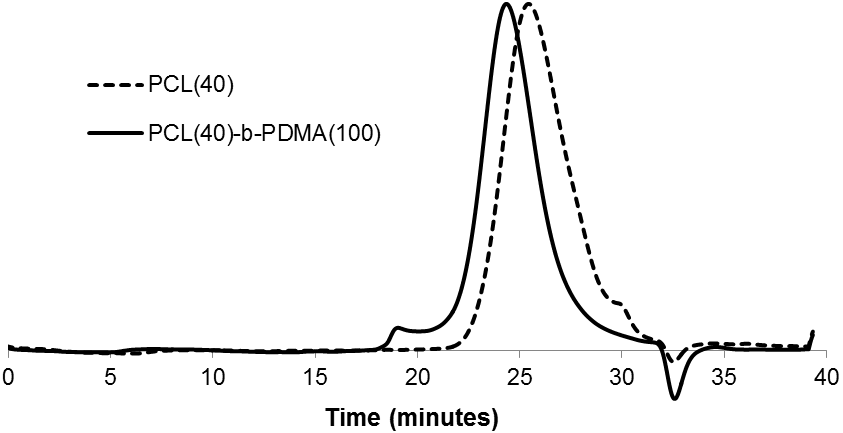
**

| Polymer | M_n_ (Da) | M_w_ (Da) | PDI | M_w_ (NMR) |
| --- | --- | --- | --- | --- |
| **1** | 28300 | 34700 | 1.24 | 4700 |
| **P1** | 39300 | 50000 | 1.27 | 20000 |

**Figure S3.** Characterization of diblock copolymer **P1** by gel permeation chromatography in DMF/LiBr (0.6 mL/min) calibrated against near monodispersed polystyrene standards. PDI of block copolymer **1** (PCL) is 1.24 and PDI of the diblock copolymer **P1** is 1.27. The M_n_ and M_w_ shown are calculated using a polystyrene standard along with the M_w_ according to the NMR spectrum (**S4**).

**
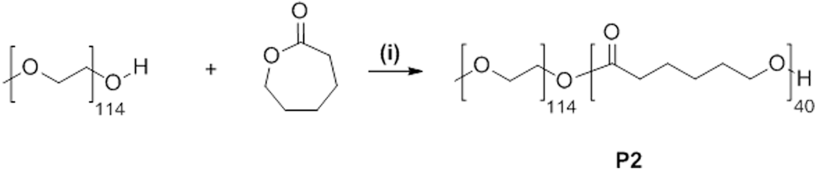
**

**Fig. S4** - Synthesis of poly(ethylene glycol)-*b*-poly(ε-caprolactone) **P2**: (i) Sn(Oct)_2_, PhCH_3_, 110 °C, 24 h.

**Figure S5.** ^1^H NMR spectrum (400 MHz, CDCl_3_) of diblock copolymer **P2**.

**
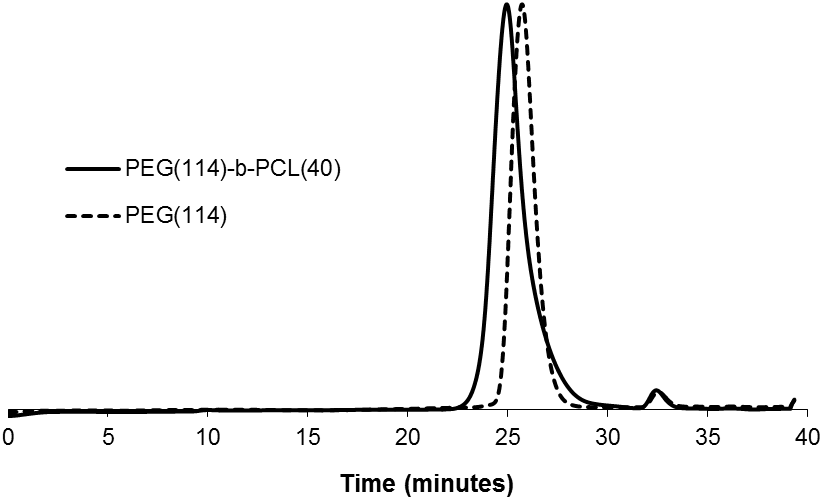
**

| Polymer | M_n_ (Da) | M_w_ (Da) | PDI | M_w_ (NMR) |
| --- | --- | --- | --- | --- |
| **3** | 29500 | 30500 | 1.03 | 5100 |
| **P2** | 35400 | 39100 | 1.10 | 10000 |

**Figure S6.** Characterization of diblock copolymer **P2** by gel permeation chromatography in DMF/LiBr (0.6 mL/min) calibrated against near monodispersed polystyrene standards. PDI of initiator polyethylene glycol **3** (PEG) is 1.03 and PDI of the diblock copolymer **P2** is 1.10. The M_n_ and M_w_ shown are calculated using a polystyrene standard along with the M_w_ according to the NMR spectrum (**S4**).


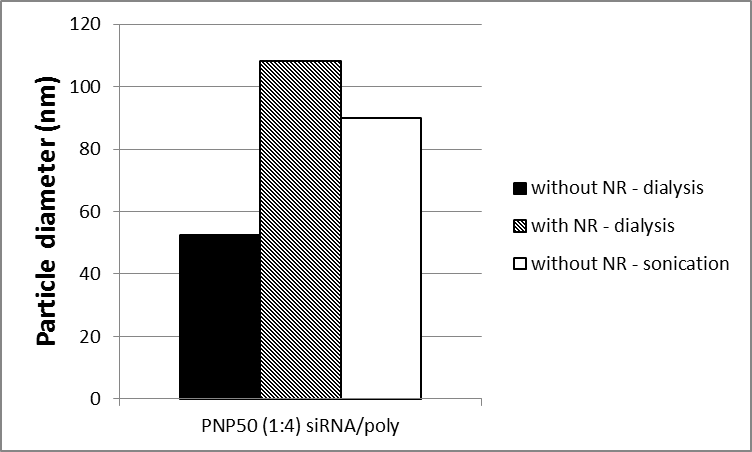


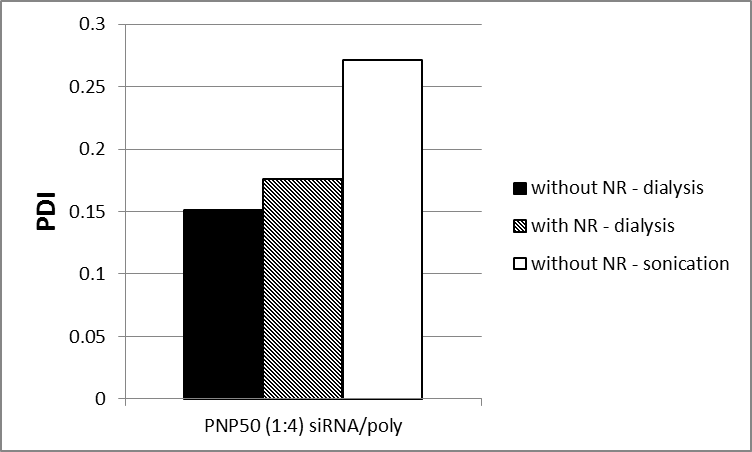


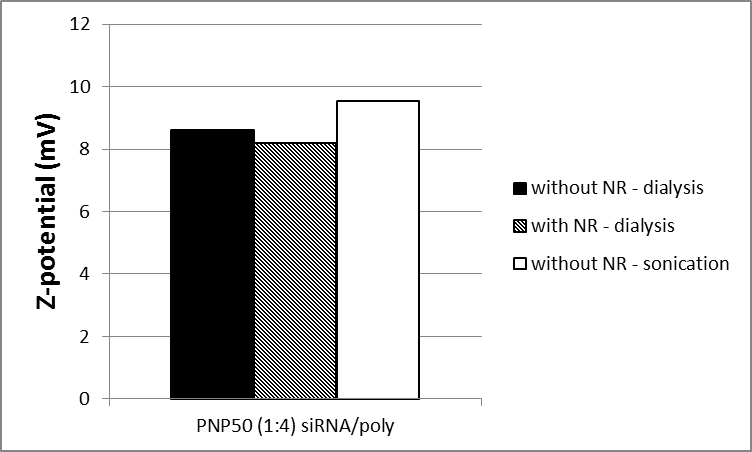


**Figure S7.** The micelles encapsulating NR were prepared in a different manner than those used for the gene knockdown experiments.

As Nile Red is a very insoluble molecule, first polymers and Nile red were co-dissolved in DMF, water was added dropwise at a very slow rate to promote micelle formation; this is when Nile red migrates to the core of the micelle. DMF is then removed by dialysis against water or a buffered aqueous solution. At last, siRNA was loaded, and the solution left to stand for 1 hour.

This method of preparing the PNPs without adding Nile red, generally gives rise to smaller size micelles, this could be due to the slower rate of micelle formation compared to the sonication method, thus, the polycaprolactone blocks are able to pack in a tighter and more organised manner. However, when Nile red is added, there is a swelling in the core of the micelle, due to the Nile red molecules taking some space within the core of the micelle, thus not letting the PCL blocks to organise so readily. The hydrodynamic diameter of such particles are however comparable with that of the particles achieved by the sonication method.

A lower polydispersity index was observed for the particles prepared using the dialysis method, compared to those prepared by sonication, giving evidence of a more monodisperse particle solution.

Despite the preparation method, in the presence or absence of Nile red, the ζ-potential values are also comparable, when the micelle is loaded with a 1:4 molar ratio siRNA/polymer.


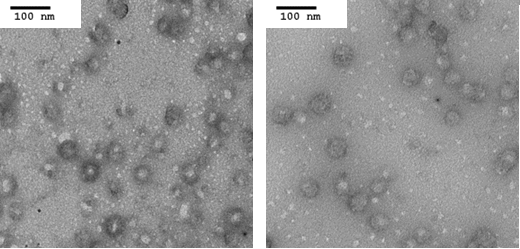


**Figure S8.** TEM images of **PNP50** mixed micelle nanoparticles. Unloaded micelles (left) and micelles loaded with a 1:4 siRNA/polymer molar ratio (right).


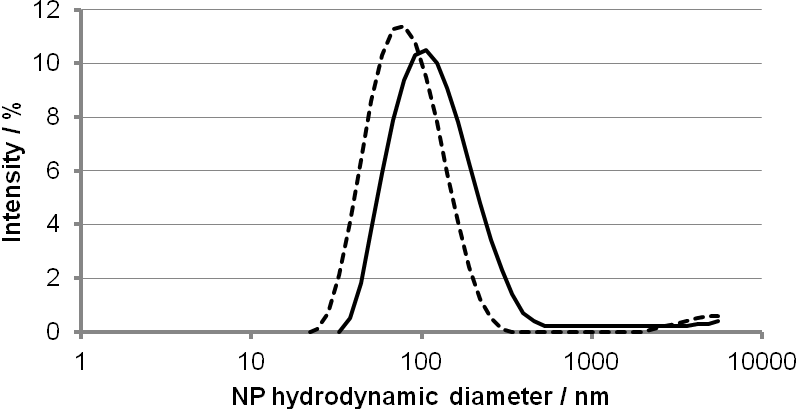


**Figure S9.** Dynamic light scattering analysis (H_2_O, 20 °C) of a 2 µM **PNP50** solution. Dotted line corresponds to the unloaded micelles with an average hydrodynamic diameter of 80 nm. Solid line corresponds to the micelles loaded with a 1:4 MR siRNA/polymer with an average hydrodynamic diameter of 108 nm.


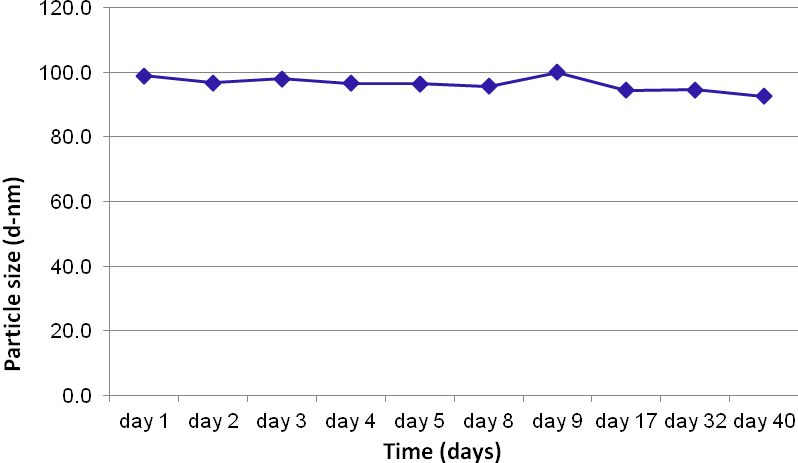


**Figure S10.** Study of the stability of **PNP50** loaded with 1:4 MR siRNA/polymer vs. time.


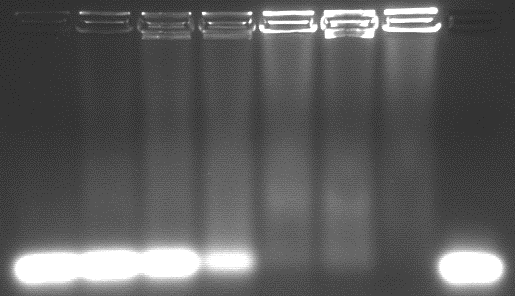

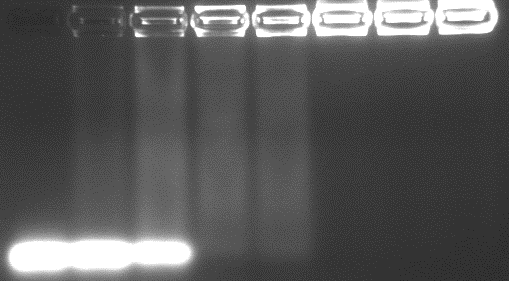

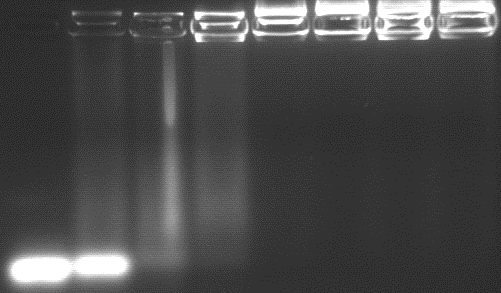

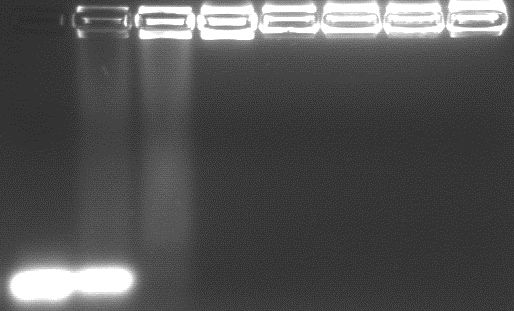

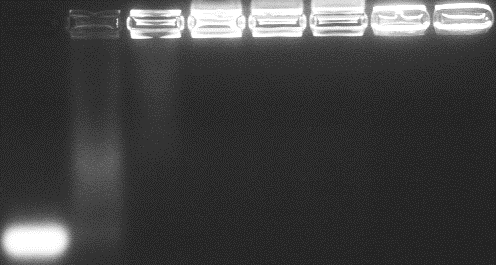

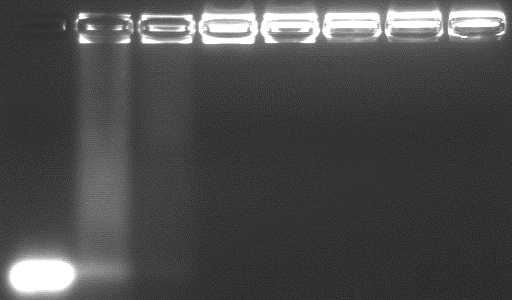

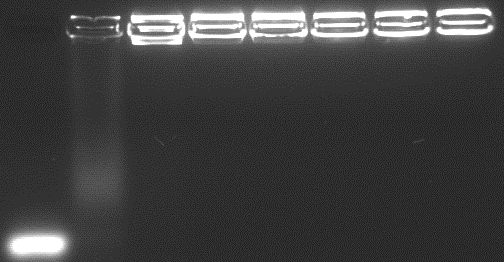

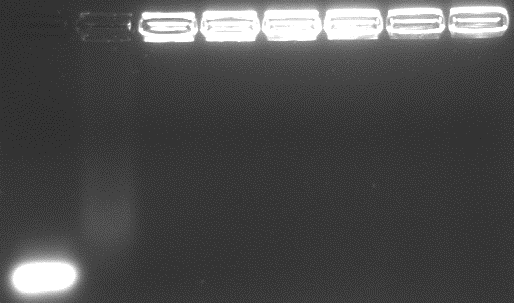


siRNA 1:1 1:2 1:4 1:6 1:8 1:10 1:12

1:1 1:2 1:4 1:6 1:8 1:10 1:12 siRNA

PNP10

PNP20

siRNA 1:1 1:2 1:4 1:6 1:8 1:10 1:12

PNP30

siRNA 1:1 1:2 1:4 1:6 1:8 1:10 1:12

PNP40

siRNA 1:1 1:2 1:4 1:6 1:8 1:10 1:12

siRNA 1:1 1:2 1:4 1:6 1:8 1:10 1:12

PNP50

PNP60

siRNA 1:1 1:2 1:4 1:6 1:8 1:10 1:12

siRNA 1:1 1:2 1:4 1:6 1:8 1:10 1:12

PNP70

PNP80

(Continued overleaf)


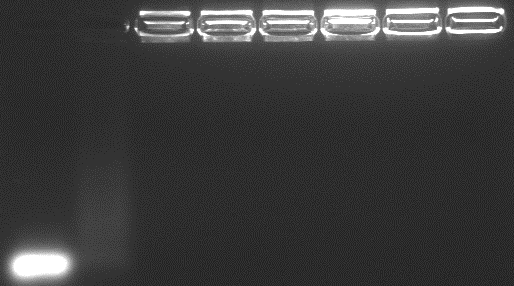


siRNA 1:1 1:2 1:4 1:6 1:8 1:10 1:12

PNP90

siRNA 1:1 1:2 1:4 1:6 1:8 1:10 1:12

PNP100


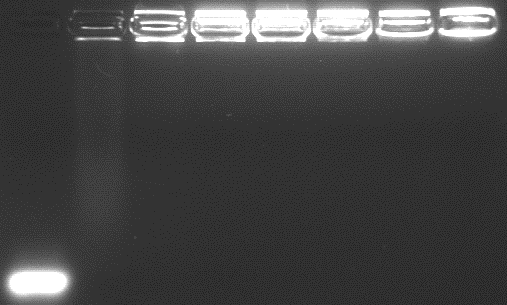


**Figure S11**. 3% Gel electrophoresis experiments for each PNP formulation with different siRNA loadings (1:1 – 1:12 siRNA/polymer molar ratios; siRNA, uncomplexed siRNA).


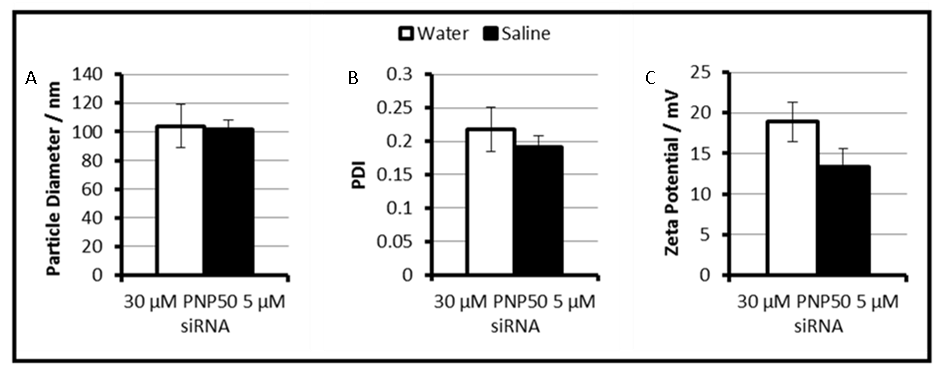


**Figure S12.** Comparison of the structure of 30 µM PNP50 loaded with 5 µM siRNA when prepared in deionised water or 0.9% saline. There is no significant difference between the size (A *[p=0.772]*) or PDI (B *[p=0.278]*) of the two preparations although the zeta potential of PNPs prepared in saline are significantly lower (C *[p=0.006]*).

PNP50 (1:4) siRNA/poly in water

PNP50 (1:4) siRNA/poly in a **10%** FBS aqueous solution

100 % FBS

**Figure S13.** Particle size comparison between 1) PNP50 in a free FBS solution, 2) PNP50 in a 10% FBS solution and 3) pure FBS.

PNP50 (1:4) siRNA/poly in water

PNP50 (1:4) siRNA/poly in a **20%** FBS aqueous solution

100 % FBS

**Figure S14.** Particle size comparison between 1) PNP50 in a free FBS solution, 2) PNP50 in a 20% FBS solution and 3) pure FBS.


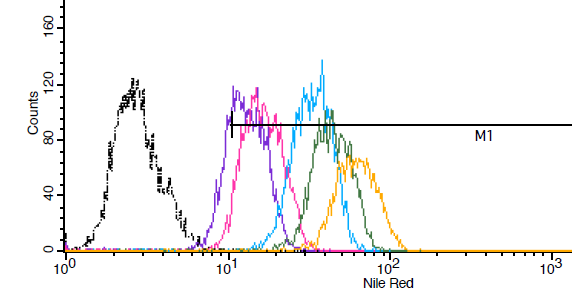


| **Key** | **Legend** |
| --- | --- |
| **---** | Untreated |
| **---** | 13 mg/l PNP50 |
| **---** | 17 mg/l PNP50 |
| **---** | 36 mg/l PNP50 |
| **---** | 48 mg/l PNP50 |
| **---** | 58 mg/l PNP50 |


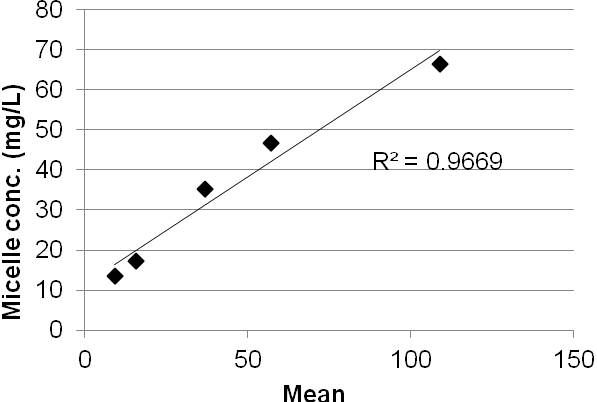


**Figure S15.** Study of the cell association on 293T cells at different concentrations of **PNP50**, loading a 1:4 MR siRNA/polymer, encapsulating Nile Red (NR). FACS histogram: black dotted area corresponds to untreated cells (blank). Purple, pink, blue, green and yellow areas are cell populations treated with 13, 17, 36, 48 and 68 mg/L concentration of **PNP50** respectively. The scatter plot shows the relationship between PNP concentration and cell association.

**
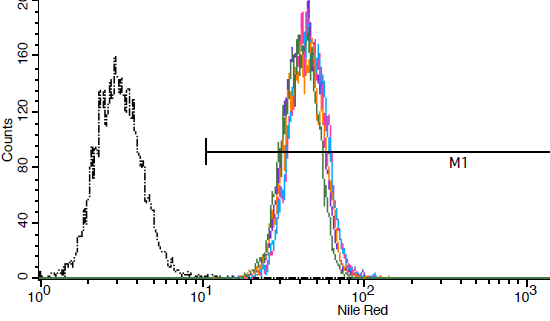
**

| **Key** | **Legend** |
| --- | --- |
| **---** | Non-treated_Blank |
| **---** | 1:2 siRNA/Poly. |
| **---** | 1:4 siRNA/Poly. |
| **---** | 1:6 siRNA/Poly. |
| **---** | 1:8 siRNA/Poly. |
| **---** | 1:10 siRNA/Poly. |

**
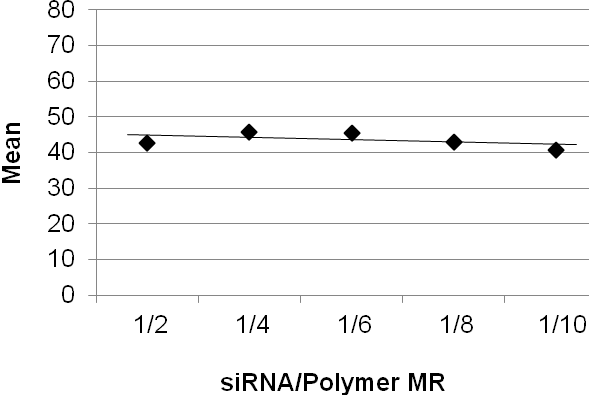
**

**Figure S16.** Study of the cell association on 293T cells of a 2 µM **PNP50** solution (encapsulating NR), at different siRNA loadings. FACS histogram: black dotted area corresponds to untreated cells. Purple, pink, blue yellow and green areas are cell populations treated with **PNP50** loaded with a 1:2, 1:4, 1:6, 1:8 and 1:10 siRNA/polymer MR respectively. The scatter plot shows the relationship between siRNA loading and cell association, giving a slope close to zero, indicating that cell association is not affected by the siRNA loading.

| 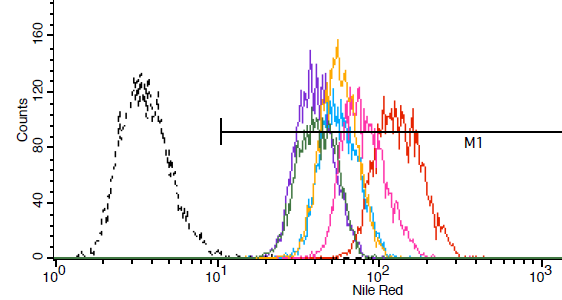**Key** | **Legend** |
| --- | --- |
| **---** | Untreated |
| **---** | PNP10 |
| **---** | PNP20 |
| **---** | PNP30 |
| **---** | PNP40 |
| **---** | PNP50 |
| **---** | PNP60 |


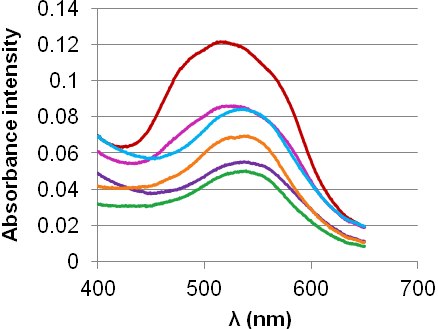


**Figure S17.** Study of the cell association on 293T cells of the polymer nanoparticles (encapsulating NR) at a concentration of 2 µM, 1:4 MR siRNA/polymer loading, and varying the proportion of cationic polymer **P1**. FACS histograms: black dotted shape corresponds to untreated cells. Red, purple, pink, blue, yellow and green indicate cell populations treated with PNP10, PNP20, PNP30, PNP40, PNP50 and PNP60 respectively. The plot below shows the Nile Red absorption of each micelle solution. Since the polymer concentration in each sample is constant, the difference in absorbance indicates that the loading capacity in the core of the micelles depends on the PNP formulations resulting in higher cell associations for those formulations with a higher loading capacity. The amount of cationic polymer within the PNP does not affect the cell association, the core packing and hence the difference in NR loading.

|  | **siGL3** | **siAGF1** | **siGL3/siAGF1** |
| --- | --- | --- | --- |
| **800 µM siRNA** | 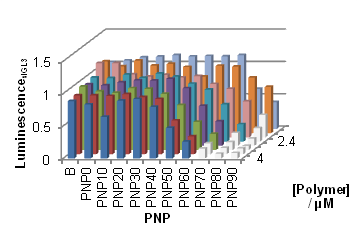 | 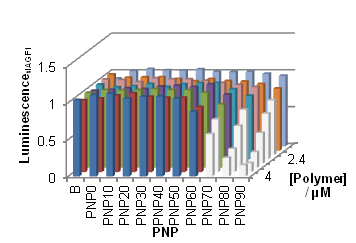 | 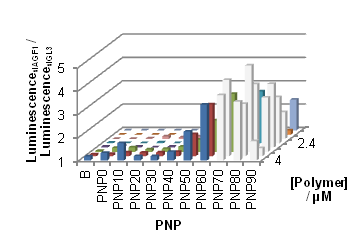 |
| **200 µM siRNA** | 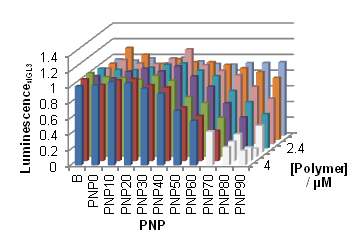 | 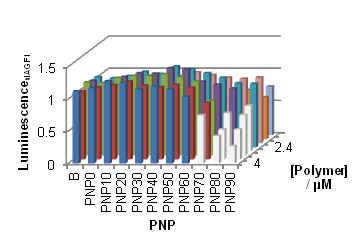 | 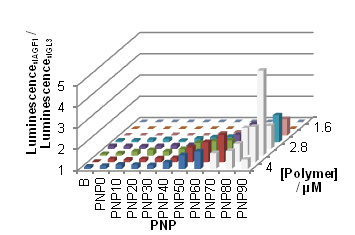 |
| **50 µM siRNA** | 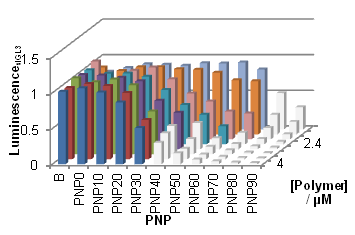 | 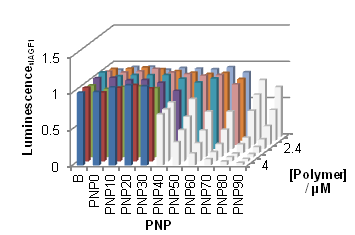 | 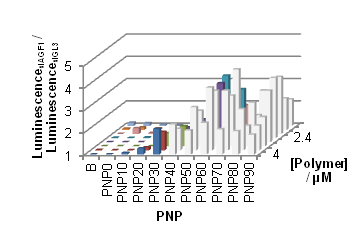 |

**Figure S18**. Table showing graphs from 3D luciferase knockdown titrations on a 293T cell line using the variables of siRNA load (rows), proportion of P1 (x axis) and total polymer concentration (z axis). Formulations which show unacceptable levels of toxicity (<80% luminescence compared to blank when treated with the control PNP) are shown by the grey columns.

|  | **siGL3** | **siAGF1** | **siGL3/siAGF1** |
| --- | --- | --- | --- |
| **1000 nM siRNA** | 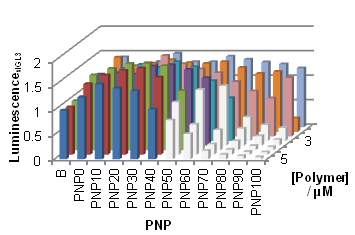 | 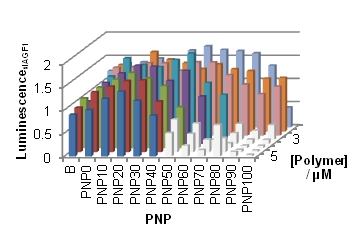 | 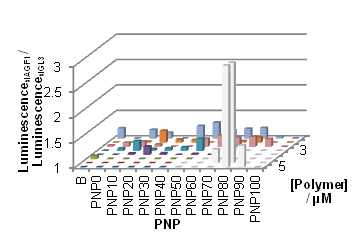 |
| **500 nM siRNA** | 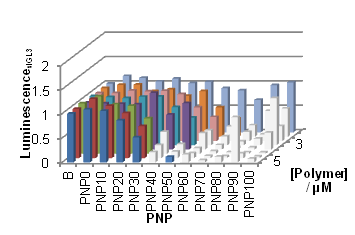 | 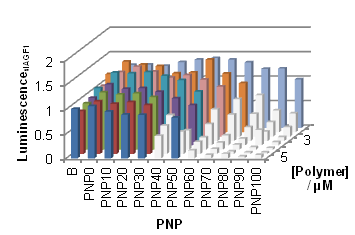 | 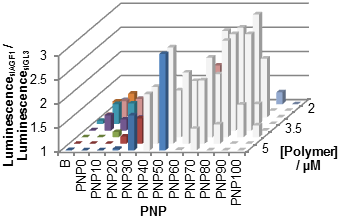 |
| **250 nM siRNA** | **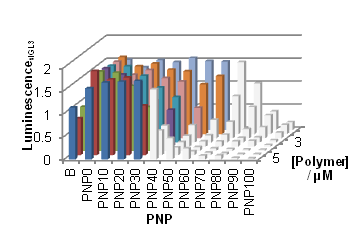** | **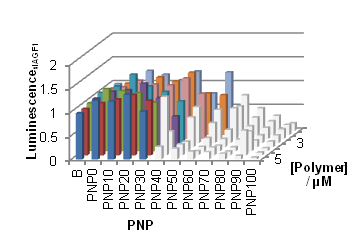** | **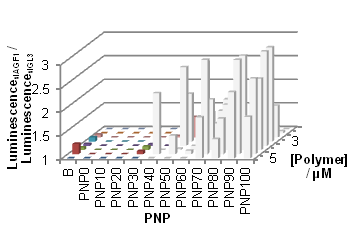** |

**Figure S19**. Table showing graphs from 3D luciferase knockdown titrations on a SKNO-1 cell line using the variables of siRNA load (rows), proportion of P1 (x axis) and total polymer concentration (z axis). Formulations which show unacceptable levels of toxicity (<80% luminescence compared to blank when treated with the control PNP) are shown by the grey columns.

PNP Particle size:

|  | **Particle diameter (nm)** | **PDI** |
| --- | --- | --- |
| **FBS free** | 101.1 | 0.215 |
| **10% FBS** | 141.3 | 0.521 |
| **20% FBS** | 119.2 | 0.626 |

FBS Particle size:

| d(nm) peak 1 | d(nm) peak 2 |
| --- | --- |
| 42.13 | 6.98 |

**Table S1.** PNP particle sizes in media

|  | **Blue Channel – DAPI** | **Red Channel – Cy5** |
| --- | --- | --- |
| **Gain** | **120** | **130** |
| **Offset** | **-21** | **-10** |
| **Power** | **39** | **27** |

**Table S2.** Laser settings for confocal microscopy

[1] W. Zhang, J. He, Z. Liu, P. Ni, X. Zhu, *Journal of Polymer Science Part A: Polymer Chemistry* **2010**, *48*, 1079-1091.

[2] H. Sun, B. Guo, R. Cheng, F. Meng, H. Liu, Z. Zhong, *Biomaterials* **2009**, *30*, 6358-6366.

[3] J. R. Harris, P. Agutter, *Journal of Ultrastructure Research* **1970**, *33*, 219-232.

[4] aM. Wilhelm, C. L. Zhao, Y. Wang, R. Xu, M. A. Winnik, J. L. Mura, G. Riess, M. D. Croucher, *Macromolecules* **1991**, *24*, 1033-1040; bK. Letchford, R. Liggins, H. Burt, *Journal of Pharmaceutical Sciences* **2008**, *97*, 1179-1190.

[5] S. Matozaki, T. Nakagawa, R. Kawaguchi, R. Aozaki, M. Tsutsumi, T. Murayama, T. Koizumi, R. Nishimura, T. Isobe, K. Chihara, *British Journal of Haematology* **1995**, *89*, 805-811.
